# Safety and Efficacy of Methylene Blue Combined with Artesunate or Amodiaquine for Uncomplicated Falciparum Malaria: A Randomized Controlled Trial from Burkina Faso

**DOI:** 10.1371/journal.pone.0001630

**Published:** 2008-02-20

**Authors:** Augustin Zoungrana, Boubacar Coulibaly, Ali Sié, Ingeborg Walter-Sack, Frank P. Mockenhaupt, Bocar Kouyaté, R. Heiner Schirmer, Christina Klose, Ulrich Mansmann, Peter Meissner, Olaf Müller

**Affiliations:** 1 Centre de Recherche en Santé de Nouna, Nouna, Burkina Faso; 2 Department of Internal Medicine VI, Clinical Pharmacology and Pharmaco-epidemiology, Medical School, Ruprecht-Karls-University, Heidelberg, Germany; 3 Institute of Tropical Medicine and International Health, Charité – University Medicine Berlin, Berlin, Germany; 4 Biochemistry Centre, Ruprecht-Karls-University, Heidelberg, Germany; 5 Institute of Medical Biometrics and Informatics, Ruprecht-Karls-University, Heidelberg, Germany; 6 Institute of Bioinformatics and Epidemiology, Medical School, Ludwig Maximilians University München, Germany; 7 Department of Tropical Hygiene and Public Health, Medical School, Ruprecht-Karls-University, Heidelberg, Germany; University of California Los Angeles, United States of America

## Abstract

**Background:**

Besides existing artemisinin-based combination therapies, alternative safe, effective and affordable drug combinations against falciparum malaria are needed. Methylene blue (MB) was the first synthetic antimalarial drug ever used, and recent studies have been promising with regard to its revival in malaria therapy. The objective of this study was to assess the safety and efficacy of two MB-based malaria combination therapies, MB–artesunate (AS) and MB–amodiaquine (AQ), compared to the local standard of care, AS-AQ, in Burkina Faso.

**Methods and Findings:**

Open-label randomised controlled phase II study in 180 children aged 6–10 years with uncomplicated falciparum malaria in Nouna, north-western Burkina Faso. Follow-up was for 28 days and analysis by intention-to-treat. The treatment groups were similar in baseline characteristics and there was only one loss to follow-up. No drug-related serious adverse events and no deaths occurred. MB-containing regimens were associated with mild vomiting and dysuria. No early treatment failures were observed. Parasite clearance time differed significantly among groups and was the shortest with MB-AS. By day 14, the rates of adequate clinical and parasitological response after PCR-based correction for recrudescence were 87% for MB-AS, 100% for MB-AQ (p = 0.004), and 100% for AS-AQ (p = 0.003). By day 28, the respective figure was lowest for MB-AS (62%), intermediate for the standard treatment AS-AQ (82%; p = 0.015), and highest for MB-AQ (95%; p<0.001; p = 0.03).

**Conclusions:**

MB-AQ is a promising alternative drug combination against malaria in Africa. Moreover, MB has the potential to further accelerate the rapid parasite clearance of artemisinin-based combination therapies. More than a century after the antimalarial properties of MB had been described, its role in malaria control deserves closer attention.

**Trial Registration:**

ClinicalTrials.gov NCT00354380

## Introduction

Early diagnosis and prompt treatment with an effective antimalarial medicine remains the mainstay of malaria control in sub-Saharan Africa (SSA) [1]. This strategy is now complicated by the increasing resistance development of *Plasmodium falciparum* to accessible and affordable first-line drugs such as chloroquine (CQ) and sulfadoxine/pyrimethamine (SP) in most countries of SSA [1]–[3].

Treatment with effective drugs as combinations has eventually become a paradigm in malaria control, with the particular aim to delay and possibly reverse the development of drug resistance [4]–[9]. Artemisinin-based combination therapy (ACT) has proved highly effective in a number of field trials [10]–[16]. However, ACT is not readily available and often prohibitively expensive in SSA [6], [9], [17], [18]. Moreover, resistance may develop also against ACT, particularly in conjunction with a long-lasting, non-artemisinin partner drug [1], [19]–[20]. Worrying evidence has already been provided that resistance to artemisinins may be selected *in vivo* by uncontrolled use of artemisinins or in combination with ineffective partner drugs [21], [22]. Finally, a number of alternative antimalarial combinations have been shown to be of at least similar efficacy compared to ACT [16], [23]–[26].

Methylene blue (MB), the first synthetic drug ever used against malaria [27], has received renewed attention in recent years [28]. MB is a subversive substrate and specific inhibitor of *P. falciparum* glutathione reductase, it inhibits the heme polymerization within the parasite's food vacuole, and prevents methaemoglobinaemia in clinical malaria [28], [29]. MB in combination with CQ has been shown to be safe in a West-African population with a high prevalence of G6PD deficiency [30]–[32]. However, due to intense CQ resistance in the study area, this combination was shown not to be sufficiently effective against malaria in young children of Burkina Faso [31], [32]. This is in accord with the impaired efficacy of ACT regimens comprising non-artemisinin partner drugs affected by high grade resistance [10], [26], [33].

Amodiaquine (AQ) has remained remarkably effective in many SSA countries despite emerging CQ resistance [11], [34]–[37]. It is considered a candidate partner drug both in ACT and in non-artemisinin drug combinations [1]. The combination MB-AQ could thus become an alternative to ACT. Likewise, MB-AS might have a role in combination treatment, particularly when considering that synergy with artemisinin derivatives has been demonstrated in a recent *in vitro* study [38].

Against this background, the safety and efficacy of MB combined with artesunate (AS) and of MB combined with AQ was studied in a controlled trial in Burkina Faso.

## Methods

The protocol for this trial and supporting CONSORT checklist are available as supporting information; see Checklist S1 and Protocol S1.

### Study area

The study was conducted in October/November 2006 in the urban research zone of the *Centre de Recherche en Santé de Nouna* (CRSN) in Nouna Health District, north-western Burkina Faso. The area is highly endemic for malaria with most clinical cases occurring during or briefly after the rainy season which lasts from June until October [39]. Although ACT has become the official first-line treatment for uncomplicated malaria in Burkina Faso since 2005, malaria control in the study area continues to be mainly based on home treatment with CQ [18], [40].

### Study design and objectives

The study was designed as a randomized controlled phase II trial. The study was open label, with blinding only for the microscopists involved. The primary objective was to investigate the safety of the combinations MB-AS and MB-AQ in children with uncomplicated falciparum malaria. The secondary objective was to determine the efficacy of these MB-based combinations in the treatment of children with uncomplicated falciparum malaria. The primary end point of the study was the incidence of observed and self-reported adverse events over the 28 days observation period. Secondary end points were: treatment outcomes until day 14 and day 28 of follow-up, i.e. adequate clinical and parasitological response (ACPR), early treatment failure (ETF), late clinical failure (LCF), late parasitological failure (LPF), fever clearance time, parasite clearance time, and hematocrit at D28 compared to baseline [41]. Study participants with LCF and those with LPF on D28 received rescue treatment with artemether-lumefantrine (CoArtem®).

### Study population

The community was informed on the project, and mothers with febrile children were invited to come to fever measurement points (specific private houses) in Nouna town (population, 25 000). Children who were considered eligible for the study (acute febrile disease, age between 6 and 10 years) were referred to the Nouna district hospital outpatient clinic for further examinations.

Inclusion criteria were: age 6–10 years, ability to swallow tablets, uncomplicated falciparum malaria (axillary temperature ≥37.5°C and ≥1,000 *P. falciparum* asexual parasites per µL blood), and written informed consent given by the parents/caretakers. The age range criterion results from the absence - at the time of the study - of a liquid, taste-masked MB formulation, thus, patients needed to be able to take coated MB tablets (Urolene Blue®, Star Pharmaceuticals, USA). Exclusion criteria were signs of severe malaria, any apparent other disease, and malaria treatment – except CQ - with western drugs and/or antibiotics with antimalarial potency during the preceding week.

### Randomization

Treatment was randomly assigned according to study identification numbers (computer-generated randomly permutated codes). A sealed envelope containing the identification numbers was opened after finalisation of the examination in the hospital and after informed consent was given.

### Study intervention

180 children were assigned to receive either MB-AS, MB-AQ, or AS-AQ, AS-AQ being the official first-line antimalarial in Burkina Faso. MB (Urolene Blue®, Star Pharmaceuticals, USA) was given at a dose of 10 mg per kilogram of body weight twice daily over three days. AS (Artesunate, Guilin Pharmaceuticals Co., Ltd., PR China) was given at a dose of 4 mg per kilogram of body weight once daily over three days. AQ (Essential Drug Store, Ministry of Health, Burkina Faso) was given at a dose of 10 mg per kilogram of body weight once daily over three days. The study drugs were administered under direct observation by study nurses (morning, at hospital) or field workers (evening, at home). In case of vomiting within the first 30 minutes the treatment was repeated. If vomiting occurred again, the patient was excluded and referred to the paediatrics department of the hospital. Children with fever ≥38.5°C received a standard dose of 10 mg per kilogram paracetamol tablets every 6 hours (Essential Drug Store, Ministry of Health, Burkina Faso) until the symptoms subsided.

### Follow-up

Follow-up of study children was for 28 days using a slightly modified version of the latest WHO protocol on antimalarial drug efficacy testing [41]. Children were included if they had at least 1,000 *P. falciparum* parasites and were followed up on days 1, 2, 3, 7, 14, and 28. Mothers and caretakers were encouraged to come back at any time between scheduled visits in case of unforeseen symptoms.

### Laboratory examinations

A finger-prick blood sample was taken on days 0, 2, 3, 7, 14, and 28, and during unscheduled visits. From this, malaria parasitaemia and haematocrit values were determined using standard CRSN procedures [39]. Thick and thin blood films were examined by two experienced laboratory technicians supervised by one of the investigators (BC). Asexual parasites were counted on thick blood films against 200 white blood cells (WBCs) and parasite density was calculated assuming an average WBC count of 10,000/µL. Slides were declared negative if no parasites were seen in 400 fields on the thick film. For quality control, a 10% random sample of blood films is regularly cross checked at the Heidelberg School of Tropical Medicine [39].

From each blood sample, an aliquot was stored on filter paper. After shipment to the Institute of Tropical Medicine and International Health Berlin, DNA was extracted using commercial kits (QIAmp, Qiagen, Germany). Differentiation of recrudescences from new infections was achieved by comparing PCR-generated *P. falciparum msp1* and *msp2* genotype patterns in matched pairs of isolates obtained on admission and on the day of reappearance of parasitaemia [42].

### Statistical analysis

The study was designed to have a statistical power of 80% in order to detect a difference in the number of adverse events of at least 20% among study groups that was significant at the five percent level. Losses to follow-up and dropouts due to other reasons were considered treatment failures in an intention-to-treat manner.

The Chi square test (Chi) was used to compare proportions, and the non-parametric Wilcoxon-Mann-Whitney test (WMW) to compare metric or ordinal data. When possible, estimates and the corresponding 95% confidence interval are given.

The closed testing procedure [43] was used to adjust for multiple testing when performing two group comparisons in a three-armed trial. In case of three groups, this procedure performs in a global test for the nullhypothesis “No difference between the three groups”. In case of a significant result, the three pairwise comparisons are performed on a five percent level. In the multiple testing situation the procedure guarantees an overall level of 5%.

Calculations were performed with SAS release 9.1 (SAS® Institute Inc, Cary, NC, USA).

### Ethical aspects

The study protocol was approved by the Ethics Committee of the Medical Faculty at Heidelberg University and the National Ethics Committee of Burkina Faso. Caretakers were asked for their written consent after having received detailed information from a study nurse about all known risks and benefits of the study through translation of a detailed research consent form into the local language,

## Results

### Enrolment and baseline characteristics of study children

**Figure 1:**
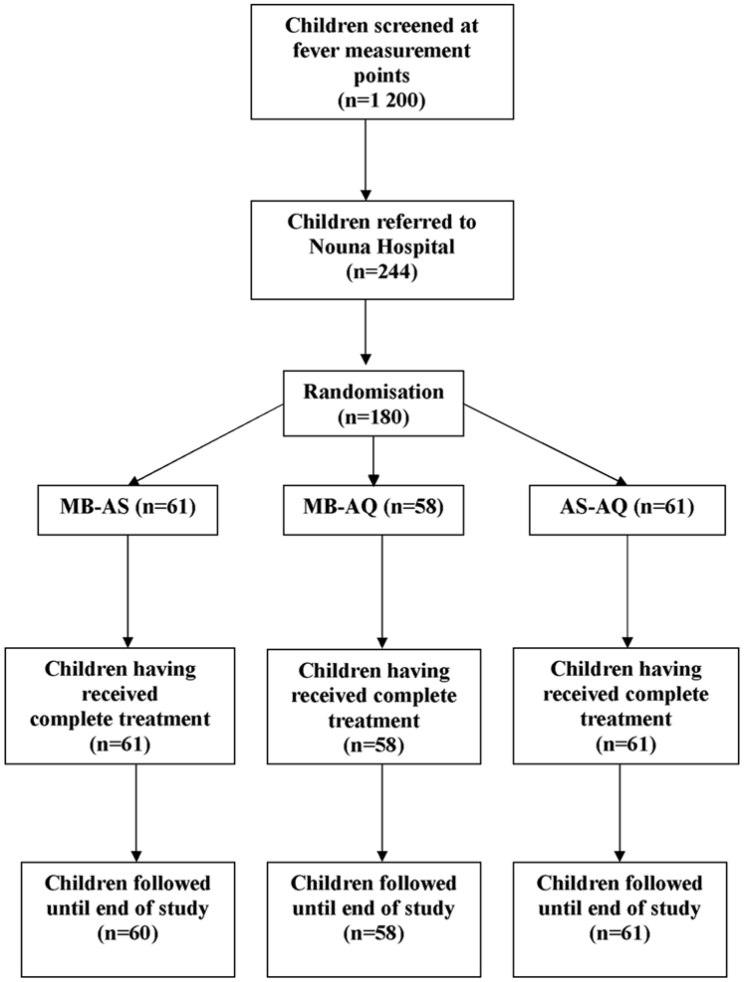
Flow chart of study patients. **Explanations:** (1) Of 244 children assessed for eligibility, 64 were excluded because of not meeting inclusion criteria; (2) In group MB-AS, one child was lost to follow-up because of family out-migration after day 7; (3) all children followed up until the end of the study were included into the analysis

Of 1 200 children seen at the fever measurement points, 244 children were referred to hospital for assessment, and of those 180 (61 MB-AS, 58 MB-AQ, 61 AS-AQ) were included into the study (Figure 1). These children form the *a priori* defined full analysis set (FAS) for the intention-to-treat analysis. There was one loss to follow-up due to out-migration from the study area (MB-AS group, after day 7). At enrolment, the demographic and clinical characteristics of the participants in the three study groups were similar, except a small but significant difference in weight (Table 1).

**Table 1:** Characteristics of study children at enrolment

Characteristic	MB-AS group (n = 61)	MB-AQ group (n = 58)	AS-AQ group (n = 61)	p-value
Female sex (%)	30 (49)	32 (55)	22 (36)	0.100
Median age in years (range)	7 (6–10)	6 (6–10)	7 (6–10)	0.512
Median weight in kg (range)	20 (14–32)	19 (13–38)	19 (13–33)	0.039
Median haematocrit in % (range)	34 (26–44)	36 (26–42)	34 (24–40)	0.246
Median number of *P. falciparum* trophozoites/µl (range)	33,000 (1,000–265,050)	28,700 (1,000–200,000)	31,000 (1,000–258,000)	0.529
Median duration of the current disease episode in days (range)	2 (1–7)	2 (1–7)	2 (1–7)	0.488
Reported CQ treatment of current disease episode (%)	6 (10)	12 (21)	10 (16)	0.256

### Safety of study drugs

There was no case of death but one serious adverse event over the 28 days follow-up period. It occurred in the AS-AQ group as a brief hospitalisation late at night on day 0 due to severe vomiting. This child was not considered a treatment failure and thus not excluded from the further analysis.

Adverse events are shown in Table 2. Compared to the two MB-receiving study groups, children in the AS-AQ group had fewer adverse events. This was mainly due to the high rates of vomiting and dysuria in both MB-receiving groups. Vomiting in the MB-receiving groups was mild and when it occurred it did so regularly between one and two hours after the intake of the study drugs. In the 81/119 (68%) children of the MB-receiving groups who experienced vomiting, its frequency was 1.5 (range 1–4) over the three days treatment period. After a thorough instruction of all mothers and caretakers to always give some food before drug administration, the frequency of vomiting decreased significantly in both MB-receiving groups (data not shown). Mild dysuria in the MB-receiving groups usually subsided after day 4. In only two cases, dysuria was reported to continue until day 7 and day 14, respectively.

**Table 2:** Adverse events in the three study groups

				p-values			
	MB-AS n = 61	MB-AQ n = 58	AS-AQ n = 61	global	MB-AS vs. MB-AQ	MB-AS vs. AS-AQ	MB-AQ vs. AS-AQ
Vomiting (%)	44 ( 72.1)	37 ( 63.8)	16 ( 26.2)	<0.001	0.329	<0.001	<0.001
Dysuria (%)	28 ( 45.9)	37 ( 63.8)	0 ( 0.0)	<0.001	0.050	<0.001	<0.001
Headache (%)	4 ( 6.6)	5 ( 8.6)	10 ( 16.4)	0.177	0.670	0.088	0.202
Pruritus (%)	0 ( 0.0)	6 ( 10.3)	0 ( 0.0)	0.001	0.010	1.000	0.010
Bronchitis (%)	4 ( 6.6)	6 ( 10.3)	7 ( 11.5)	0.624	0.457	0.343	0.843
Diarrhoea (%)	1 ( 1.6)	3 ( 5.2)	3 ( 4.9)	0.534	0.285	0.309	0.949
Others (%)	11 ( 18.0)	5 ( 8.6)	18 ( 29.5)	0.014	0.132	0.137	0.004

### Efficacy of study drugs

Efficacy results are given in Table 3. No ETF was observed. By day 14, the ACPR rate after PCR-based correction for recrudescence was significantly lower in the MB-AS group (87%) compared to the two AQ-receiving groups (100%).

**Table 3:** Efficacy outcomes in the three study groups

				p-values			
Outcome	MB-AS n = 61	MB-AQ n = 58	AS-AQ n = 61	global	MB-AS vs. MB-AQ	MB-AS vs. AS-AQ	MB-AQ vs. AS-AQ
Parasitaemia							
- until D2	7 (11.5%)	37 (63.8%)	13 (21.3%)	<0.001	<0.001	0.081	<0.001
- until D3	1 (1.6%)	10 (17.2%)	3 (4.9%)				
ETF	0 (0.0%)	0 (0.0%)	0 (0.0%)	1.000	1.000	1.000	1.000
LPF( D14)							
- without PCR-based correction	6 (9.8%)	0 (0.0%)	1 ( 1.6%)	0.011	0.014	0.052	0.327
- with PCR-based correction	4 (6.6%)	0 (0.0%)	0 (0.0%)	0.018	0.047	0.042	1.000
LPF( D28)							
- without PCR-based correction	24 (39.3%)	11 (19.0%)	16 (26.2%)	0.043	0.015	0.123	0.344
- with PCR-based correction	13 (21.3%)	3 (5.2%)	9 (14.8%)	0.038	0.010	0.346	0.083
LTF (D14)							
- without PCR-based correction	6 (9.8%)	0 (0.0%)	0 (0.0%)	0.002	0.014	0.012	1.000
- with PCR-based correction	4 (6.6%)	0 (0.0%)	0 (0.0%)	0.018	0.047	0.042	1.000
LTF (D28)							
- without PCR-based correction	20 (32.8%)	1 (1.7%)	5 (8.2%)	<0.001	<0.001	<0.001	0.107
- with PCR-based correction	10 (16.4%)	0 (0.0%)	2 (3.3%)	<0.001	0.001	0.015	0.164
ACPR (D14)							
- without PCR-based correction	49 (80.3%)	58 (100.0%)	60 (98.4%)	<0.001	<0.001	0.001	0.327
- with PCR-based correction	53 (86.9%)	58 (100.0%)	61 (100.0%)	<0.001	0.004	0.003	1.000
ACPR (D28)							
- without PCR-based correction	17 (27.9%)	46 (79.3%)	40 (65.6%)	<0.001	<0.001	<0.001	0.094
- with PCR-based correction	38 (62.3%)	55 (94.8%)	50 (82.0%)	<0.001	<0.001	0.015	0.030

Similarily, by day 28, the rate of ACPR without correction was significantly lower in the MB-AS group (28%) compared to the groups MB-AQ (79%) and AS-AQ (66%). During the 28 days of follow-up, exclusively new genotypes, i.e. re-infections, were more frequent with MB-AS (34.4%; 21/61) than with MB-AQ (15.5%; 9/58; p = 0.02) or AQ-AS (16.4%; 10/61; p = 0.02). After PCR-based correction for recrudescence, the ACPR rates increased to 62% (MB-AS), 95% (MB-AQ), and 82% (AS-AQ), differences being significant among all groups (table 3).

Fever was cleared rapidly in all three groups; only two patients remained febrile until day 2 in the MB-AS group, and two until day 1 in the MB-AQ group. However, there were significant differences in parasite clearance among study groups (table 3): While in the MB-AS group only 7/61 patients had parasites (range 40–200/µL) on day 2, and this was further reduced to 1/61 (100/µL) by day 3, in the MB-AQ group, 46/58 patients had parasites (range 50–5,000/µL) on day 2 and this was reduced to 10/58 (range 40–120/µL) by day 3. Patients receiving AS-AQ showed an intermediate pattern of parasitaemia prevalence on day 2 (14/61, range 40–500/µL) and day 3 (3/61, all 40/µL). Parasite clearance differences were significant when comparing the MB-AS and MB-AQ groups (p<0.001) as well as the MB-AQ and AS-AQ groups (p<0.001), and were close to significance when comparing MB-AS with AS-AQ (p = 0.081).

There were no significant changes in median haematocrit values over time and among groups (data not shown).

## Discussion

Treatment with the MB-based combinations was associated with significantly more adverse events compared to AS-AQ, and this was caused by the frequent occurrence of vomiting and dysuria, symptoms already known to be associated with MB tablets [44]. However, these events were always mild and self-limiting. Importantly, vomiting was shown to be much reduced by administering MB together with food which should be the clear advice in further studies.

Urolene Blue® is a taste-masked MB tablet used for other indications in patients who are able to swallow tablets. There is currently no paediatric taste-masked formulation on the market and the provision of non-masked liquid MB solutions or crushed tablets dissolved in water is not recommendable as the drug has a strong bitter-metallic taste [31], [32]. Therefore, a taste-masked MB syrup has recently been developed by our group together with the group of Prof. Breitkreutz from the University of Düsseldorf in Germany which currently undergoes clinical testing. Such a paediatric MB formulation will allow further clinical studies with MB-based combinations in children below five years of age, the main risk group of malaria in SSA [1].

Two main results with regard to the efficacy of the MB-based combinations are provided by the present trial. First, the efficacy of MB-AQ in the study area is high, and significantly higher than it is the case for the standard ACT regimen. With 95% ACPR after 28 days of follow-up, the efficacy of MB-AQ is in the range of various ACT regimens in SSA [10]–[13], [15], [16]. This high cure rate of MB-AQ might partially result from the comparatively advanced age of study participants and, consequently, immunity contributing to parasite elimination. This, however, would not explain the superiority of MB-AQ over AQ-AS, and, also, suggests that the striking cure rate of MB-AQ is unlikely to be explained by the efficacy of AQ alone. A recent trial on AQ in older patients (median age, 4.2 years) from western Burkina Faso demonstrated a PCR-corrected ACPR rate of only 82% after 28 days of follow-up [37]. Similarily, data from 117 children aged five years or less and treated in Nouna town in 2005 with AQ showed an ACPR rate after PCR-based correction for recrudescence at day 28 of only 61% (Mandi et al., unpublished). Finally, preliminary results on the high efficacy of MB monotherapy in semi-immune adults with falciparum malaria provide further evidence for a substantial contribution of MB to the efficacy of MB-based combination therapies (Müller et al., unpublished).

At 82% ACPR, the standard regime AS-AQ surprisingly did worse than expected and than it has been reported previously from other places in West Africa [10], [16]. It is currently not clear if this finding reflects local differences in AQ resistance, a real inferiority of AS-AQ compared to MB-AQ, or both. Although the AQ used for this study was taken from a local source, we are confident that it is of good quality. Drugs from the essential drug store of the Ministry of Health are regularly quality controlled and a recent study on the quality of malaria drugs in the Nouna Health District provided no evidence for quality problems with AQ (Maike Tipke, unpublished).

Secondly, although performing rather poor in terms of overall efficacy, MB-AS achieved a more rapid clearance of *P. falciparum* parasites than the other two treatment regimes. This provides evidence in humans for a clinically relevant synergy between an artemisinin derivative and MB, which has already been shown *in vitro* [38]. Adding MB to ACT may thus be an option to further speed up the rapid parasite clearance conferred by the artemisinin derivatives [4], [6]–[8].

Likely related to the short elimination half-life of both MB and AS [32], [45], re-infections were more common in children treated with this combination than in patients receiving treatment containing AQ. Also, rapid parasite clearance in MB-AS may interfere with the development of sustained immune mechanisms preventing re-infections. Similar findings have been observed in northern Ghana where the fastest regimen in parasite clearance, SP-AS, produced the highest proportion of re-infections [46].

As MB is both available and affordable, the combination MB-AQ would be an interesting alternative antimalarial regimen when shown to be safe and effective in larger multi-centre phase III studies. Such studies are now planned for in the frame of a public-private-partnership by our group. Apart from the potential benefit of another effective antimalarial regimen contributing to a healthy competition of different regimens on the market, MB can be considered a re-emerging antimalarial with the potential to be a valuable partner drug in various ACT and non-artemisinin drug combinations.

In conclusion, MB-AQ is a promising alternative drug combination against falciparum malaria in SSA. Moreover, MB has the potential to further accelerate parasite clearance when used in ACT. Further studies are needed to define the possible roles of MB in malaria treatment.

## Supporting Information

Checklist S1 CONSORT Checklist
(0.06 MB DOC)

Protocol S1 Trial Protocol
(0.20 MB DOC)
