# Supplementary material for: Safety and Efficacy of Methylene Blue Combined with Artesunate or Amodiaquine for Uncomplicated Falciparum Malaria: A Randomized Controlled Trial from Burkina Faso
Source: PLoS One. 2008 Feb 20;3(2):e1630. doi: 10.1371/journal.pone.0001630 (PMC2238815; doi:10.1371/journal.pone.0001630)
Supplement: Protocol S1 — Trial Protocol (0.20 MB DOC) [file pone.0001630.s002.doc]

Version of July 11, 2006

Safety and efficacy of methylene blue combined with artesunate or amodiaquine for malaria treatment in children of Burkina Faso:

a pilot study in the frame of the A8 Project of the SFB 544

**Principal investigators:** PD Dr. med. Olaf Mueller, MD, MPH

Department of Tropical Hygiene and Public Health

Ruprecht-Karls-University Heidelberg

INF 324, 69120 Heidelberg, Germany

Tel.: +49 6221 56 5035 Fax: +49 6221 56 5039

E-mail: [olaf.mueller@urz.uni-heidelberg.de](mailto:olaf.mueller@urz.uni-heidelberg.de)

Dr. Peter Meissner, MD, MSc Trop Paed

Department of Tropical Hygiene and Public Health

Ruprecht-Karls-University Heidelberg

INF 324, 69120 Heidelberg, Germany

Tel.: +49 6221 56 5035 Fax: +49 6221 56 5039

E-mail: [peter.meissner@urz.uni-heidelberg.de](mailto:peter.meissner@urz.uni-heidelberg.de)

**Biometrician:** Prof. Dr. rer. nat. Ulrich Mansmann

Chair of Biometry and Bioinformatics

IBE, Medical School, LMU München

Marchioninistr. 15, 81377 Muenchen, Germany

Tel: +498970954491 Fax: +498970957491

E-mail: [mansmann@ibe.med.uni-muenchen.de](../mansmann@ibe.med.uni-muenchen.de)

**Trial coordinator:** Dr. Mandi Germain, MD

Nouna Health Research Centre

P.O. Box 34, Nouna, Burkina Faso

Tel.: +226 537055 Fax: +226 537055

E-mail: [mandi_germain@yahoo.fr](../mandi_germain@yahoo.fr)

**Sponsor:** Ruprecht-Karls-University Heidelberg, Klinikum,

SFB 544, INF 365, 69120 Heidelberg, Germany

**Members of study team:** Dr. Bocar Kouyaté, MD, MPH

Director, Nouna Health Research Centre

P.O. Box 34, Nouna, Burkina Faso

Tel.: +226 537055 Fax: +226 537055

E-mail: [Bocar.crsn@fasonet.bf](mailto:Bocar.crsn@fasonet.bf); [bkouyate@hotmail.com](mailto:bkouyate@hotmail.com)

Dr. sc. hum. Boubacar Coulibaly

Head of laboratory, Nouna Health Research Centre

P.O. Box 34, Nouna, Burkina Faso

Tel.: +226 537055 Fax: +226 537055

E-mail: [boubacoulibaly@hotmail.com](mailto:boubacoulibaly@hotmail.com)

Prof. Dr. med. Ingeborg Walter-Sack

Department of Internal Medicine VI, Clinical Pharmacology and Pharmacoepidemiology

Ruprecht-Karls-Univ. Heidelberg, 69120 Heidelberg, Germany

Tel.: + 49 6221 56 8742 Fax: +49 6221 56 4642

E-mail: [ingeborg.walter-sack@med.uni-heidelberg.de](mailto:ingeborg.walter-sack@med.uni-heidelberg.de)

Prof. Dr. med. Heiner Schirmer

Biochemie-Zentrum Heidelberg

Ruprecht-Karls-University Heidelberg

INF 328, 69120 Heidelberg

Tel.: +49 6221 54 4165/4175 Fax: +49 6221 54 5356

E-mail: [heiner.schirmer@gmx.de](mailto:heiner.schirmer@gmx.de)

Prof. Dr. rer. nat. Heiko Becher

Department of Tropical Hygiene and Public Health

Ruprecht-Karls-University Heidelberg

INF 324, 69120 Heidelberg, Germany

Tel.: +49 6221 56 5031 Fax: +49 6221 56 5948

E-mail: Heiko.Becher@urz.uni-heidelberg.de

# Protocol summary

Title: Safety and efficacy of methylene blue combined with artesunate or amodiaquine for malaria treatment in children of Burkina Faso: a pilot study in the frame of the A8 Project of the SFB 544

Design: Single-centre, randomized controlled study in children with uncomplicated falciparum malaria in the Nouna Health District, north-western Burkina Faso

Phase: Phase II

Objectives: The primary objective of this trial is to study the safety of the combination methylene blue (MB)-artesunate (AS) and MB-amodiaquine (AQ) given over three days in 3-4 years old children with uncomplicated falciparum malaria in an area of high malaria transmission intensity compared to the safety of a three days AS-AQ regimen. Secondary objectives are to investigate the efficacy of MB-AS and MB-AQ.

Population: Children aged 3-4 years with uncomplicated malaria from Nouna town.

Sample size: N= 180 (n=60 for each group).

Treatment: The participants in the MB-AS group will receive orally twice daily 10mg/kg MB combined with once daily 4mg/kg AS over 3 days. The participants in the MB-AQ group will receive orally twice daily 10mg/kg MB combined with once daily 10mg/kg AQ over 3 days. The participants of the comparator group will receive a 3 day regimen of once daily oral AS (4mg/kg) combined with once daily AQ (10mg/kg).

Endpoints: The primary endpoint is the number of adverse events (AE) after drug intake until day 28. Secondary endpoints are the number of serious adverse events (SAE), adequate clinical and parasitological response (ACPR) rate on day 28, clinical and parasitological failure rates on day 3, 7, 14 and 28, changes in haemoglobin/haematocrit until day 28, and fever and parasite clearance time.

Study duration and dates: The study will be implemented during the rainy season of the year 2006 (July - October).

# Flow chart for study treatments

Day 0

Day 1

Day 2

Day 3

Day 4

Day 7

Day 14

Day 28

**Blood sample**

**Blood sample**

**Blood sample**

**Blood sample**

**Blood sample**

Inclusion/Exclusion

Informed Consent

Treatment 1

**Examination 1**

**Blood sample**

Treatment 2

Treatment 3

**Examination 2**

Treatment 6

**Examination 4**

Treatment 4

**Examination 5**

**Examination 7**

**Examination 6**

Treatment 5

**Examination 3**

3 days regimen MB-AS or MB-AQ or AS-AQ

# Abbreviations

| MB | Methylene Blue |
| --- | --- |
| AS | Artesunate |
| AQ | Amodiaquine |
| SAE | Serious adverse event |
| AE | Adverse event |
| SSA | Sub-Saharan African |
| CRF | Case Report Form |
| CRSN | Nouna Health Research Centre |
| ACPR | Adequate clinical and parasitological response |
| ETF | Early treatment failure |
| LCF | Late clinical failure |
| LPF | Late parasitological failure |

# Definitions

| Term | Definition |
| --- | --- |
| Baseline values | Directly after the written informed consent a blood sample will be taken for the baseline measurements (laboratory baseline values). |
| Age | The age is calculated using the date of informed consent and date of birth. If the day of the birth date is unknown, it is set to 15. If day and month are missing the 1st of July is considered as the date of birth. |
| D0, D1, D2, D3, D7, D14 and D28 | For the definition of days the latest WHO Definition is used (WHO 2003).  D0= calendar day of the first treatment, D1= first calendar day after the calendar day of first treatment. This definition is used for the endpoints ETF, LCF, LPF, ACPR and fever or parasite clearance time. D7, D14, D28 is defined as the 7^th^, 14^th^ and 28^th^ day after first drug intake, with a tolerance of –1 and +3 days. |
| ETF | - Danger signs or severe malaria on D1, D2 or D3 + parasitemia - Parasitemia on D2 > D0 count irrespective of axillary temperature - Parasitemia on D3 ≥ 25% of count on D0 |
| LCF | - Development of danger signs or severe malaria after D3 in the presence of parasitemia, without meeting any of the criteria of ETF - Presence of parasitemia + axillary temperature ≥37.5°C on any day from D4 to D28, without previously meeting any criteria of ETF |
| LPF | - Presence of parasitemia on any day from D7 to D28 + axillary temperature <37.5°C, without previously meeting any of the criteria of ETF |
| ACPR | - No parasites on D28 irrespective of axillary temperature without previously meeting any of the criteria of ETF, LCF or LPF |
| Fever clearance | Time between fever at inclusion and first temperature < 37.5°C |
| Parasite clearance | Time between inclusion and time where no more parasites are detectable |
| Recrudescence | Re-emerging of the same *P. falciparum* strain from D14 to D28 according to PCR genotyping |
| Reinfection | New infection from D14 to D28 according to PCR genotyping |
| AEs | The adverse event (=AE) defined in chapter 12 is used, but blue urine is expected for all children in the MB-AS and MB-AQ treatment groups. Only AEs after initiation of study treatment will be tabulated. |
| SAE | The definition of serious adverse event (SAE) is given in chapter 11 |
| Haemolysis | Any drop in haemoglobin (Hb) of >2.5 g/dl within 24 hours |
| Severe acute haemolysis | Hb ≤ 5 g/dl or received blood transfusion according to clinical judgement of study physician. |
| Severe acute neurological disorder | Acute change in consciousness  ≥ 2 convulsions |

# Introduction and study rationale

## Malaria and malaria control in Africa

Malaria remains the most important parasitic disease and is globally responsible for 300-500 million fever episodes and 1.5-2.7 million deaths per year (WHO 1997). The greatest burden of malaria is in sub-Saharan African (SSA) where it has been estimated that 40% of fever episodes are caused by malaria and where children regularly experience several malaria episodes a year (Brinkmann and Brinkmann 1991, Müller et al. 2001). Most malaria deaths occur in young children of rural SSA areas with little access to health services (WHO 1997, Greenwood et al. 1987, Snow et al. 1999, Müller et al. 2003).

In SSA, the basis of malaria control is early diagnosis and prompt treatment with effective antimalarial drugs. This strategy is now complicated through the increasing development of resistance by *Plasmodium falciparum* to existing and affordable first-line drugs such as chloroquine and sulphadoxine-pyrimethamine (Trapé 2001). Parasitological resistance to sulfadoxine-pyrimethamine has reached already 20-40% in parts of East Africa and increasing resistance has been described also for West Africa (Mockenhaupt et al. 2005). The situation is likely to become critical in a couple of years (White et al. 1999). If antimalarial multidrug resistance emerges in SSA at the speed observed in south-east Asia, the development of alternative drug regimens is of overriding public health importance.

Treating malaria with a combination of effective drugs is therefore becoming a new paradigm in malaria control, with the particular aim to delay and possibly reverse the development of drug resistance through different mechanisms (Nosten and Brasseur 2002). Artemisinin drugs in combination with a variety of partner drugs have proved highly effective in a number of field trials (International Artemisinin Study Group 2002). Although the costs for artemisinin combination therapy (ACT) are currently still higher than the price for chloroquine and pyrimethamine-sulfadoxine regimens, more and more African countries are currently switching their antimalarial first line treatment to ACT. So far no resistance to ACT has been described in vivo. Most research focuses on the use of artesunate combined with currently used standard drugs such as amodiaquine and sulphadoxine-pyrimethamine. There is evidence that combinations improve efficacy without increasing toxicity, however cure rates vary widely and depend on the level of resistance of the partner drug. New compounds with novel mechanisms of action and a short half-life to prevent resistance are therefore urgently needed to protect such ACT. The combination of the two short acting drugs methylene blue and artesunate or the combination of methylene blue with the long-acting and still sufficiently effective AQ ought to be considered as a possible solution to this problem in SSA (Akoachere et al. 2005, Barker et al. 2005).

## MB in the treatment of malaria

MB (Methylthioniniumchlorid) is a water-soluble dye used for a very long time in industry and medicine. In medicine, it can be administered both orally and parenterally. In humans, the drug is rapidly and widely distributed throughout the body (DiSanto and Wagner 1972). It is generally crossing the blood-brain barrier, but according to animal data i. v. administration initially seems to lead to much higher concentrations in the central nervous system than oral administration (Peter et al. 2000). The drug is well absorbed from the gastrointestinal tract, maximal plasma concentrations are reached after 1-2 hours following oral administration, and the plasma half-life is around 10-12h hours (own unpublished data). Renal excretion of MB is the main elimination pathway: 53-97% of an oral dose is excreted in unchanged or leuco-form in the urine over a 5 day period (DiSanto and Wagner 1972, Peter et al. 2000). In sheep, the 24 hours LD_50_ after a single i. v. MB administration was 42.3 mg/kg (95% CI: 37.3-47.9); methaemoglobin production was minimal with doses as high as 50 mg/kg, and no significant haematological changes were seen up to 4 weeks after a total dose of 30 mg/kg was administered i. v. over 18 hours (Burrows 1984). From this study it was concluded that the dosage of i. v. MB may safely be increased up to at least 15 mg/kg in the therapy of severe methaemoglobinaemia in humans. However, in one older publication i. v. doses of MB exceeding 7 mg/kg have been reported to cause distressing symptoms, such as restlessness, fine tremor, nausea and chest pain. Grayish blue discolouration of the skin, resembling cyanosis, but with normal values for pO2, and haemolytic anaemia was observed if i. v. doses of 15 mg/kg or more were given to children (Goluboff and Wheaton 1961). In these case reports no significant methaemoglobinaemia was observed. However, MB was well tolerated in oral doses of up to 17 g (5 x 100-200 mg per day) during initial observations in malaria therapy in adults (Guttmann and Ehrlich 1891). Moreover, no significant side effects besides some mild dysuria were reported during treatment of 40 young children, including infants, with malaria in Brasil with oral doses of 20-50 mg/kg/day MB over several days to weeks (Ferreira 1893). It thus appears as if MB given orally is safe even in higher doses compared to intravenous application. This idea is supported by some evidence for significant differences in organ distribution leading to major differences in pharmacokinetics between oral and intravenous MB administration (Peter et al. 2000). Finally, all persons taking MB develop blue urine over the following days.

MB has already been successfully used against malaria some 100 years ago (Guttmann and Ehrlich 1891, Ferreira 1893, Ehrlich 1913). Its further use against human malaria is not well documented, thus only observational data support its efficacy, particularly in malaria cases found refractory to quinine (Dale 1956). The drug was no longer used against human malaria after other effective and non-toxic drugs appeared on the market (e.g. CQ). However, MB has been shown to be the most effective of its analogues in inhibiting the growth of *P. falciparum* in culture, and its antimalarial efficacy in the mouse model is achieved at concentrations similar to the ones used in the treatment of methaemoglobinaemia in humans (Vennerstrom et al. 1995). MB appears to act similar to 4-aminoquinoline antimalarials by preventing the polymerization of heme, which is produced during the digestion of host cell cytosol in the parasite food vacuole, into hemozoin. In the past, the drug has also been used against gastrointestinal and urogenital infections and against rheumatism. MB is a registered drug in most countries for a number of still very relevant indications. It is mainly known for its efficacy in the treatment of methaemoglobinaemia both in adults and in children (e.g. intoxication with nitrite or a number of methemoglobin-inducing drugs), and for the treatment and prevention of ifosfamide-induced encephalopathy in cancer treatment. In the case of methaemoglobinaemia and for prevention of ifosfamide-induced encephalopathy, the dosage is 1-2 mg/kg i. v. 2-6 times per day (Mansuri and Lurie 1993, Küpfer et al. 1994, Zulian et al. 1995, Aeschlimann et al. 1998, Pelgrims et al. 2000). As methaemoglobin formation has been reported as a potential serious complication of falciparum malaria in Africa, the use of MB as an antimalarial may help to prevent tissue anoxia (Anstey et al. 1996). MB is furthermore locally used for diagnostic purposes, for photodynamic therapy in tumour treatment, against skin and mucosa infections, topically against ulcera (10% solution), and may be even be beneficial against sepsis (Orth et al. 1995, Galili et al. 1997).

The interest in MB as an antimalarial drug was reactivated when *P. falciparum* glutathione reductase was identified as a target of the drug in the parasite (Färber et al. 1998, Sarma et al. 2003, Schirmer et al. 2003). MB inhibits the glutathione reductase of *P. falciparum* but not the human glutathione reductase at therapeutically used concentrations (Böhme et al. 2000, Kanzok et al. 2000, Davioud-Charvet et al. 2003).

There was some evidence that MB, being a glutathione reductase inhibitor, might potentiate the activity of CQ or even reverse CQ resistance (Ginsburg 2002, Sarma et al. 2003). Therefore the safety and efficacy of MB against uncomplicated malaria in humans was first tested in the combination with CQ. This drug combination was found to be safe even in G6PD deficient adults and children (Mandi et al. 2005, Meissner et al. 2005). The combination was however not sufficiently effective against uncomplicated falciparum malaria in young children when given at a dose of 12 mg/kg over three days (Meissner et al. 2005). In a subsequent dose finding study in a comparable population of young children, a significantly better efficacy of MB against uncomplicated malaria was demonstrated when 3-6 times higher MB doses were given over three days (unpublished data). Although there was a significant reduction especially in early treatment failure rates, an unacceptable high rate of recrudescence most likely due to the high background resistance of the local parasites against CQ was observed.

Therefore the ideal partner drug to MB still needs to be identified. Two studies have recently shown a synergistic effect of the drugs MB and AS in vitro and in rhesus monkeys (Akoachere et al. 2005, Barker et al. 2005). As both drugs are short acting and no resistance has been demonstrated so far, AS-MB appears to be a promising drug combination. However, to be useful under real life conditions in SSA such a combination needs to be highly effective in a three days regimen. Monotherapy with AS for three days achieved only low cure rates (between 20% and 50% on day 28) in nonimmune populations outside SSA (Borrmann 2003). However, only few studies have studied the efficacy of shorter regimens of AS monotherapy in SSA. While five day regimens of AS monotherapy reached rather high cure rates in semiimmune populations of Tanzania and Nigeria, a three days regimen conducted in 50 children aged 4-15 years with uncomplicated malaria in a hyperendemic area of Gabon had cured only 72% of children by day 28 (Borrmann 2003). However, in this study, the day 14 cure rate was still 92% and children developed mainly asymptomatic parasitaemia afterwards.

AQ alone has been shown to be still sufficiently effective in many areas of SSA where chloroquine is failing (Müller et al. 1996, Greenwood et al. 2005). The combination of AS-AQ has been shown to be highly effective in SSA (International Artemisinin Study Group 2002). AS-AQ together with artemether-lumefantrine have been chosen as the two new official first-line treatments of uncomplicated malaria in Burkina Faso since the year 2006. Thus, comparing the two MB-based combinations MB-AS and MB-AQ with the standard of care AS-AQ is important to judge the safety of these regimens in Burkina Faso and to provide first data on the comparable efficacy.

With effect of May 2006, the World Health Organisation has officially recommended to no longer using malaria monotherapy in the treatment of uncomplicated falciparum malaria (Nelson 2006).

# Trial objectives and purpose

The purpose of the study is to investigate the safety and efficacy profile of the combinations MB-AS and MB-AQ in comparison with AS-AQ in African children with uncomplicated falciparum malaria.

The primary objective of this study is:

- To investigate the safety of the combinations MB-AS and MB-AQ in children with uncomplicated malaria caused by *Plasmodium falciparum*

Justification: MB, AS, AQ and AS-AQ have been shown to be safe in the target population. However, to treat children with the combination of MB-AS or MB-AQ is new. Thus, all adverse events of these new combinations are of interest. The comparison with AS-AQ, the new official first-line treatment for uncomplicated malaria in Burkina Faso, will help to judge the safety of MB-AS and MB-AQ in the study population. This trial should give the necessary information about the safety of the combination MB-AS and/or MB-AQ for judgment and planning of future phase III trials.

The secondary objective of this study is:

- To investigate the efficacy of MB-AS and MB-AQ

Justification: The combination MB-chloroquine has been shown to be effective against malaria in the Nouna study population. However a high rate of recrudescences was observed due to high background resistance to CQ (Meissner, unpublished). Therefore alternative drug combinations with MB need to be identified. There is now sufficient evidence from in vitro and animal models for existence of synergy between the two short acting drugs MB and AS (Akoachere et al. 2005, Barker et al. 2005). Thus AS seems to be an ideal partner drug to MB but it remains to be shown that MB-AS is sufficiently safe and effective when given over a period of three days. Alternatively, the combination MB-AQ could still be sufficiently effective even in areas of high resistance against chloroquine. This trial should give the necessary information about the efficacy of the combinations MB-AS and MB-AQ for judgment and planning of future phase III trials.

# Definition of outcomes

Primary endpoint:

- Incidence of observed and self-reported non-serious adverse events over the 28 days observation period (definition chapter 11)

Secondary endpoints:

- Incidence of serious adverse events (definition: chapter 11) over the 28 days observation period
- ACPR rate until D28
- Early treatment failure (ETF) rate
- Late clinical failure (LCF) rate at D14 and D28
- Late parasitological failure (LPF) rate at D14 and D28
- Fever clearance time
- Parasite clearance time
- Change in haematocrit after 2, 3, 7, 14 and 28 days compared to baseline

*Justification:* SAE rates and clinical and parasitological failure rates as well as fever and parasite clearance times are standard endpoints to judge the safety and efficacy of antimalarial drugs. This information will be relevant to plan future phase III trials.

# Design of the trial

Single-centre, randomized, controlled phase II trial in children aged 3-4 years with uncomplicated falciparum malaria.

Randomisation and blinding

After creation of computer generated envelopes, 180 children aged 3-4 years with uncomplicated falciparum malaria will be randomized to one of the three treatment groups: MB-AS (3 day regimen), MB-AQ (3 day regimen), AS-AQ (3 day regimen).

Due to the coloration of urine through MB, blinding of patients will not be possible. However, all lab technicians examining the blood samples for malaria parasites and other study relevant aspects will be blinded to treatment regimen.

Duration of the trial

The study will become implemented between July and October 2006.

# Selection and withdrawal of subjects

Sample size

180 children (60 children in each of the three different treatment regimens) will be enrolled into the study. The sample size of 60 per arm enables us to demonstrate a difference in AE rates of 10 % with 90% power and at a 0.05 significance level.

Inclusion criteria

- 3-4 year (36-59 months) old children
- Between 10 kg and 22.5 kg body weight
- Ability to swallow tablets
- Uncomplicated malaria caused by *P. falciparum*
- Asexual parasites ≥ 2000/µl and ≤ 200000/µl
- Axillary temperature ≥ 37.5°C
- Burkinabe nationality
- Informed consent

Exclusion criteria

- Complicated or severe malaria
- Any apparent significant disease
- Anaemia (haematocrit < 21%)
- Treated in the same trial before
- Antimalarial treatment prior to inclusion (last three days), except children having been treated with chloroquine

Withdrawal criteria

Participants may be withdrawn from the study at their guardians request and any change in the patient’s condition which in the investigator’s opinion, for reasons of safety or ethics, precludes further participation in the trial. In case of vomiting after the repeated drug intake (one repetition only) of the first drug ingestion, the participant will be withdrawn from the study. Subjects withdrawn from the study during the first 3 days will be replaced.

# Treatment of subjects and clinical study procedures

Description of the treatments

All patients in the MB-AS group will receive for 3 days twice daily (morning and evening) 10mg/kg MB accompanied by once daily (morning) 4mg/kg artesunate (= 4mg/kg total daily dose).

MB will be given according to the following weight table:

10,0 – 16,0 kg = 130mg = 2 tablets per treatment = 2 x 2 tablets /day

16,5 – 22,5 kg = 195mg = 3 tablets per treatment = 2 x 3 tablets/ day

AS will be given according to the following weight table:

10,0 – 16,0 kg = 50mg = 1 tablet per treatment = 1 tablet per day

16,5 – 22.5 kg = 100mg = 2 tablets per treatment = 2 tablets per day

AQ will be given according to the following weight table:

10,0 – 16,0 kg = 100mg = 1 tablet per treatment = 1 tablet per day

16,5 – 22.5 kg = 200mg = 2 tablets per treatment = 2 tablets per day

Product description

MB (Urolene Blue®, Star Pharmaceuticals, USA); 1 tablet Urolene Blue® = 65mg)

AS (Artesunate®, Guilin Pharmaceuticals Co., Ltd., China; 1 tablet Artesunate® = 50mg)

AQ (taken from the essential drug store of the Ministry of Health, Burkina Faso; 1 tablet amodiaquine=100mg)

Concomitant treatments

Patients having received antimalarial treatment prior to inclusion (last three days) will be excluded except children having been treated with chloroquine.

All children having fever ≥ 38.5°C will receive standard doses of paracetamol (= acetaminophen; 10 mg/kg) until symptoms subside (taken from the essential drug stock of the Ministry of Health, product description: paracetamol tablets or syrup).

All indicated drugs are allowed, except other western antimalaria drugs, antibiotics with antimalarial efficacy, acetanilide and phenacetin, nalidixic acid, niridazole, nitrofurantoins and sulphonamides (or any other drug that might induce haemolysis in G6PD deficient individuals) from beginning of treatment until end of follow-up.

Prior and concomitant illnesses

History of prior illnesses will become investigated and recorded. All relevant concomitant illnesses will be recorded based on the examination of the study physicians at D0, D1, D2, D3, D7, D14 and D28.

Procedures for monitoring subject compliance

Drug intake will be directly monitored by a study nurse or a field worker for the whole treatment period, supervised by the study physicians.

Visits and treatment schedule

After identification of febrile children in the specified age group in the community, enrolled children are treated and supervised as outpatients according to the study flow explained in chapter 3. Children are examined at regular intervals (D0, D1, D2, D3, D7, D14 and D28) by a study physician. In case of not coming to the clinic on respective examination days, children will be visited at home by field staff. Parents/guardians are instructed to return to the hospital in case of worsening of their child’s clinical condition at any time during the follow-up period.

Day 0

- Presentation of the febrile child at one of the fever measurement points
- Informed consent
- Baseline status (weight, temperature)
- Examination 1 (at the Nouna district hospital)
- Questioning for inclusion/exclusion criteria
- Capillary blood sample for a thin and thick blood film (malaria) and haematocrit
- 1 filter paper is kept for genotypic determination of G6PD deficiency
- 1 filter paper is kept to distinguish between recrudescence and reinfection according to PCR during follow-up
- Treatment 1 (before 14:00) – MB-AS or MB-AQ or AS-MB
- Treatment 2 (evening) – MB

Day 1

- Examination 2 (morning)
- Treatment 3 (morning) – MB-AS or MB-AQ or AS-MB
- Treatment 4 (evening) – MB

Day 2

- Examination 3 (morning)
- Capillary blood sample (parasitemia and haematocrit)
- Treatment 5 (morning) – MB-AS or MB-AQ or AS-MB
- Treatment 6 (evening) – MB

Day 3

- Examination 4 (morning)
- Capillary blood sample (parasitemia and haematocrit) (morning)

Day 7, 14 and 28

- Examination 5, 6 and 7 respectively (morning)
- Capillary blood sample for parasitemia and haematocrit

Other time periods

- Phaenotypical G6PD determination and further investigations according to study physicians judgement will take place at any day between D0 and D28 in case of haemolysis
- 1 filter paper is kept to distinguish between recrudescence and reinfection according to PCR during follow-up (D7, 14, 28 and any other day the child returns for examination between D3 and D28)

Clinical management of (serious) adverse events

Study physicians and emergency medications will be available 24h to deal with SAEs. Additional laboratory tests are available 24h if needed and a standard blood transfusion service is established at the hospital (e.g. in case of haemolysis).

# Assessment of primary endpoint

Specification of parameter

- Number and proportion of patients with at least one observed or self-reported adverse event (definition below)

Methods of data collection

Data on the occurrence of adverse events (severity, seriousness, causality), concomitant illnesses and concomitant drugs will be collected through direct continuous active clinical surveillance (including laboratory results) of all study subjects. All children will be examined by the responsible study physician at D0, D1, D2, D3, D7, D14 and D28.

# Assessment of secondary endpoints

Specification of parameters

- Number and proportion of patients with at least one serious adverse event:
  1. acute haemolysis (definition: haemoglobin ≤ 5 g/dl and haematocrit ≤ 15%, or received blood transfusion according to clinical judgement of study physician)
  2. Other serious adverse events (definition below)
- ACPR will be determined according to the standard WHO protocol (WHO 2003)
- Clinical and parasitological failure rates on day 3, 7, 14 and 28 will be determined according to the standard WHO protocol (WHO 2003)
- Time between inclusion and disappearance of fever
- Time between inclusion and clearance of parasites
- Change in the haematocrit after 2, 3, 7, 14 days and 28 days compared to baseline

Methods of data collection

The following laboratory methods will be employed:

Haematocrit will be measured with a centrifuge. Parasites will be diagnosed with standard techniques by light microscopy. Differentiation of recrudescences from new infections will be achieved by comparing PCR-generated *msp1* and *msp2* genotype patterns in matched pairs of isolates obtained on admission and precisely on the day of reappearance of parasitaemia.

Adverse event (AE) definition

The term “adverse event” covers any sign, symptom, syndrome, illness that appears or worsens in a subject during the period of observation in the clinical trial and that may impair the well-being of the subject. The term also covers laboratory findings or results of other diagnostic procedures that are considered to be clinically relevant.

No causal relationship with the study medication is implied by the use of the term “adverse event”. Adverse events fall into the categories non-serious and serious.

Serious adverse event (SAE) definition (GCP)

A serious adverse event is defined as any untoward medical occurrence at any dose:

(1) results in death (2) is life-threatening (3) requires inpatient hospitalisation or prolongation of existing hospitalisation (4) results in persistent or significant disability/incapacity (5) is a congenital anomaly / birth defect
(“Life-threatening” refers to an event in which the patient was at risk of death at the time of the event; it does not refer to an event which hypothetically might have caused death if it was more severe.)

Classification of severity

Mild: The adverse event does not interfere with the routine activities. The patient may experience slight discomfort.

Moderate: The adverse event interferes with routine activities. The patient may experience significant discomfort.

Severe: The adverse event makes it impossible to perform routine activities. The patient may experience intolerable discomfort or pain.

Classification of causality

Unrelated, possibly related, probably related, definitely related; cannot be assessed, for instance due to lack of information.

Unexpected adverse reaction (UAR) definition (EU Directive 2001/20/EC of 4 April 2001)

An adverse reaction, the nature or severity of which is not consistent with the applicable product information.

Period of observation of adverse events

This is the period between the informed consent and the final examination on day 28.

Documentation of adverse events and serious adverse events

All adverse and serious adverse events will be recorded on specific forms. The adverse event form contains a detailed description of the adverse event (event, beginning and duration, severity, outcome, causality to the study medication, specific therapy), serious adverse event (yes/no), and date and signature of the attending physician.

For serious adverse events (SAEs) a description of the SAE and consequences for the trial are documented.

Reporting of serious adverse events

Serious adverse events have to be reported by the attending physician to one of the principal investigators within 24 hours or not later than the next working day. The SAE is reported to both of independent Ethic Committees (Heidelberg and Burkina Faso).

# Statistics

Sample size calculation

When the sample size in each group is 54, a 5% level Chisquare test will have 80% power to detect a difference in proportions by a variance of proportions of 0,009 and an average proportion of 0.183. This szenario is met by the assumption that the rate of adverse events is 25% in the AQ+AS group as well as in the AQ +MB group, and 5% in the AS+MB group.

After a global Chisquare test for the Nullhypothesis "No difference between the three treatment groups" pairwise differences between groups will be tested by two group Chisquare tests. All 4 tests are on the 5% level and no adjustment of the significance level is needed because of the closed testing principle. On closed testing procedures with special reference to ordered analysis of variance. 95% confidence intervals will be calculated for the AE rates. Descriptive statistics will use minimum, 25% quantile, mean, median, 75% quantile, maximum für continuos values and tabulation for categorical values.

# Trial monitoring and auditing

Existence of patients (using the list of expense allowance) and SAEs will be monitored 100%. The informed consents will be checked.

The sponsor is allowed to perform an audit of the investigator site in Nouna. The corresponding documents and data are to be provided by the audited units.

# Ethical and legal aspects

The trial will be conducted in accordance with local laws and the internationally established principles for Good Clinical Practice which have their origin in the Declaration of Helsinki of the World Medical Association (WMA).

Ethics Committee Review

The protocol will be submitted to the ethics committees in Heidelberg and Burkina Faso for review and approval. Protocol amendments will be signed by the PIs and submitted to the ethics committees.

# Documentation and use of trial findings

Data management will be performed at the CRSN in Nouna. Double data entry will be performed. Queries will be formulated and resolved in the CRSN in Nouna in co-operation with the Department of Tropical Hygiene and Public Health, University of Heidelberg.

Statistical advice on analyzing the data will be provided by Prof. H. Becher (University of Heidelberg) and Prof. U. Mannsmann (LMU University of Munich).

# Trial duration and dates

The study will be implemented between July and October 2006. Training of field staff and pilot testing of methods will take place 4 weeks before the start of the study.

Early termination of the trial

Reasons that may require the termination of the trial include the following: (1) Incidence of adverse events indicates a potential health hazard caused by the study treatment. (2) It appears that participant’s enrolment or study logistics are unsatisfactory. (3) External evidence that makes it necessary to terminate the trial.

# References

Aeschlimann C, Küpfer A, Schefer H, Cerny T (1998) Comparative pharmacokinetics

of oral and intravenous ifosfamide/mesna/methylen blue therapy. Drug metabolism and disposition 26, 883-890

Akoachere M, Buchholz K, Fischer E, Burhenne J, Haefeli WE, Schirmer RH, Becker K

In vitro assessment of methylene blue on chloroquine sensitive and resistant plasmodium falciparum strains reveals synergistic action with artemisinins (in press)

Anstay NM, Hassanali MY, Mlalasi J, Manyenga D, Mwaikambo ED (1996) Elevated

levels of methaemoglobin in Tanzanian children with severe and uncomplicated malaria. Trans R Soc Trop Med Hyg 90, 147-151

Barker TL, Gettayacamin M, Hansukjariya P, Van Gessel Y, Melendez V, Teja-Isavadham P,

Vennerstrom J, Miller RS, Orth CK (2005) Efficacy of methylene blue and artesunate as combination antimalarial therapy in a rhesus monkey model of uncomplicated blood-stage malaria. Clinical Pharmacology & Therapeutics ; 77 (2), 59

Böhme CC, Arscott LD, Becker K, Schirmer RH, Williams CH (2000) Kinetic

characterization of glutathione reductase from the malarial parasite Plasmodium falciparum – comparison with the human enzyme. J Biol Chem 275, 37317-37323

Borrmann S, Adegnika AA, Missinou MA, Binder RK, Issifou S, Schindler A, Matsiegui PB Kun JFJ, Krishna S, Lell B, Kremsner PG (2003) Short-course artesunate treatment of

uncomplicated P. falciparum malaria in Gabon. Antimicrobial Agents and Chemotherapy 47: 901-904

Brinkmann U and Brinkmann A (1991) Malaria and health in Africa: the present

situation and epidemiological trends. Tropical Medicine and Parasitology 42, 204-213

Burrows GE (1984) Methylene blue: effects and disposition in sheep. J vet Pharmacol

Therap 7, 225-231

Dale HH (1956) Introduction. In Himmelweit F, Marquardt M, Dale H (ed): The

collected papers of Paul Ehrlich. Pergamon Press, London

Davioud-Charvet E, Delarue S, Christophe Biot C, et al. Double-headed prodrugs as

antimalarials. A p. falciparum glutathione reductase inhibitor conjugated with an antimalarial 4-anilinoquinoline. J Med Chem (in press)

DiSanto AR, Wagner JG (1972) Pharmacokinetics of highly ionized drugs II:

methylene blue – absorption, metabolism and excretion on man and dog after oral administration. J Pharm Sci 61, 1086-1090

Ehrlich P (1913) Chemotherapeutics: scientific principles, methods, and results. The

Lancet ii, 445-451

Färber PM, Arscott LD, Williams CH Jr, Becker K, Schirmer RH (1998)

Recombinant Plasmodium falciparum glutathione reductase is inhibited by the antimalarial dye methylene blue. FEBS Lett 423:311-314

Ferreira C (1893) Sur l´emploi du bleu de méthylène dans la malaria infantile. Revue de Thérapeutique Médico-Chirugicale 1893, 488-525

Galili Y, Kluger Y, Mianski Z, et al. (1997) Methylene blue – a promising modality in

sepsis induced by bowel perforation. Eur Surg Res 29, 390-395

Ginsburg H. (2002) A double-headed pro-drug that overcomes chloroquine resistance.

Trends Parasitol. 18, 103

Goluboff N, Wheaton R (1961) Methylene blue induced cyanosis and acute hemolytic

anemia complicating the treatment of methemoglobinemia. J Pediatr 58, 86

Greenwood B, Bradley A., Greenwood A, Byass P, Jammeh K, Marsh K, Tulloch F,

Oldfield F, Hayes R (1987) Mortality and morbidity from malaria among children in a rural area of The Gambia. Transactions of the Royal Society of Tropical Medicine and Hygiene 81, 478-486

Greenwood BM, Bojang K, Whitty JM, Targett GA (2005) Malaria. *The Lancet* **365**, 1487-98

Guttmann P, Ehrlich P (1891) Über die Wirkung des Methylenblau bei Malaria.

Berliner Klinische Wochenschrift, 39, 953-956

International Artemisinin Study Group (2002) Artesunate combinations for treating

uncomplicated malaria: a prospective individual patient data meta-analysis. In: The Cochrane Library, Issue 2, Oxford

Kanzok SM, Schirmer RH, Türbachova I, Iozef R, Becker K (2000) The thioredoxin

system of the malaria parasite P. falciparum. Glutathione reduction revisited. J Biol Chem 275, 40180-40186

Küpfer A, Aeschlimann C, Wermuth B, Cerny T (1994) Prophylaxis and reversal of

ifosfamide encephalopathy with methylene blue. The Lancet 343, 763-764

Mandi G, Witte S, Meissner P, Coulibaly B, Mansmann U , Rengelshausen J et al. (2005)

Safety of the combination of chloroquine and methylene blue in healthy adult men with G6PD deficiency from rural Burkina Faso. Tropical Medicine and International Health 10, 32-38

Mansuri A, Lurie AA (1993) Concise Review: Methemoglobinemia. American

Journal of Hematology 42, 7-12

Meissner P, Mandi G, Witte S, Coulibaly B, Mansmann U, Rengelshausen J, Schiek W, Jahn

A, Sanon M, Tapsoba T, Walter-Sack I, Mikus G, Burhenne J, Riedel KD, Schirmer H, Kouyaté B, Müller O (2005) Safety of the methylene blue plus chloroquine combination in the treatment of uncomplicated falciparum malaria in young children of Burkina Faso. Malaria Journal 4: 46

Mockenhaupt FP, Ehrhardt S, Dzisi SY, Bousema JT, Wassilew N, Schreiber J, Anemana SD, Cramer JP, Otchwemah RN, Sauerwein RW, Eggelte TA, Bienzle U (2005) A randomized, placebo-controlled, double-blind trial on sulfadoxine-pyrimethamine alone or combined with artesunate or amodiaquine in uncomplicated malaria. Tropical

Medicine and International Health 10, 512-520

Müller O, Boele van Hensbroek M, Jaffar S, Drakeley C, Okorie C, Joof D, Pinder M,

Greenwood B. A randomized trial of chloroquine, amodiaquine, and pyrimethamine-sulfadoxine in Gambian children with uncomplicated malaria. Tropical Medicine and International Health, 1: 124-132 (1996)

Müller O, Becher H, Baltussen A, Ye Y, Diallo D, Konate M, Gbangou A, Kouyate

B, Garenne M (2001) Effect of zinc supplementation on malaria morbidity among Westafrican children: a randomized double-blind placebo-controlled trial. British Medical Journal, 322, 1567-1572

Nelson R (2006) WHO requests halt of sales of monotherapy drugs for malaria. Lancet

Infectious Diseases 6, 132

Nosten F, Brasseur P (2002) Combination therapy for malaria – the way forward?

Drugs 62, 1315-1329

Orth K, Rück A, Stanescu A, Beger HG (1995) Intraluminal treatment of inoperable

oesophageal tumours by intralesional photodynamic therapy with methylene blue. The Lancet 345, 519-520

Pelgrims J, De Vos F, Van den Brande J, et al. Methylene blue in the treatment and

prevention of ifosfamide-induced encephalopathy: report of 12 cases and review of the literature. British Journal of Cancer 82, 291-294

Peter C, Hongwan D, Küpfer A (2000) Pharmacokinetics and organ distribution of

intravenous and oral methylene blue. Eur J Clin Pharmacol 56, 247-250

Sarma GN, Savvides SN, Becker K, Schirmer M, Schirmer RH, Karplus PA (2003)

Glutathione reductase of the malarial parasite Plasmodium falciparum: Crystal structure and inhibitor development. J Mol Biol (in press)

Schirmer RH, Coulibaly, B, Schiek W et al. (2003) Methylene blue in the treatment of

malaria – past and presence, Redox Report (in press)

Snow RW, Craig M, Deichmann U, Marsh K (1999) Estimating mortality, morbidity

and disability due to malaria among Africa`s non-pregnant population. Bulletin of the World Health Organisation 77, 624-640

Trape J (2001) The public health impact of chloroquine resistance in Africa. American
 Journal of Tropical Medicine and Hygiene 64 (supplement), 12-17

Vennerstrom JL, Makler MT, Angerhofer CK, Williams JA (1995) Antimalarial dyes

revisited: xanthenes, azines, and thiazines. Antimicrobial Agents and Chemotherapy 39, 2671-2677

White NJ, Nosten F, Looareesuwan S, et al. (1999) Averting a malaria disaster. The

Lancet 353, 1965-1967

WHO (1997) World malaria situation in 1994. Weekly Epidemiological Record 72,

269-292

WHO (2003) Assessment and monitoring of antimalarial drug efficacy for the treatment of

uncomplicated falciparum malaria WHO/HTM/RBM/2003.50

Zulian GB, Tullen E, Maton B (1995) Methylene blue for ifosfamide-associated

encephalopathy. The New England Journal of Medicine 332, 1239-1240

Annex:

Safety and efficacy of methylene blue combined with artesunate or amodiaquine for malaria treatment in children of Burkina Faso:

a pilot study in the frame of the A8 Project of the SFB 544

**Research Consent Form**

**Research Consent Form for Parents and Caretakers**

Purpose

We are conducting a study to find out which of three different drug regimens will be the best in curing malaria in children of Burkina Faso. Also we want to find out if the three therapies are comparable in tolerability. As your child has been diagnosed with malaria, we like to ask you to let your child participate on this study under our close supervision. About 180 children will participate in this study.

Procedure

If you agree to take part in this study, we will decide by a lottery system, whether your child will receive the combination of artesunate and methylene blue or the combination of amodiaquine and methylene blue or the combination of artesunate and amodiaquine over three days. During a 28 days follow-up period, we will invite you for regular visits to the hospital (day 7, day 14, day 28), where your child will be examined carefully including taking blood samples. The total amount of blood during the study will not exceed 10ml. All expenses will of course be covered by the study. You would need to return to the hospital with your child if it falls ill again during this 28 day study period.

Risks and discomfort

All malaria drugs used in this study are known for a long time. They are usually well tolerated, but in some persons, changes in the blood level can occur. This is why we will check your child's blood regularly and would treat your child appropriately in case of a blood level change or any other side effect. In the worst case this can imply blood transfusion.

Benefits

If you agree to participate on this study, your child will be examined and treated free of charge for its malaria disease and any other medical problem occurring during the four weeks study period. The benefit for your community will be the possible development of a new effective and affordable malaria drug.

General information

Your child's personal data will be handled confidentially. If you have any question about this study, we will be glad to tell you more. The participation is voluntary and you can refuse to take part in this study at any time and for any reason. If you begin this study, but decide to quit later, you and your child will not face any disadvantage.

The parent/caretaker agrees to what has been read.

………………….……………………………………..

Date and signature (or fingerprint) of parent/caretaker

…………………………………………………………..

Date and name and signature of responsible study nurse
